# Improving Diagnosis of Hepatitis C Virus Infection Using Hepatitis C Core Antigen Testing in a Resource-Poor Setting

**DOI:** 10.1590/0037-8682-0253-2020

**Published:** 2021-02-10

**Authors:** Ayswarya Kannan, Lalitha Biswas, Anil Kumar, Jessy Kurian, Anjaly S.Nair, Parasmal Suresh, Shine Sadasivan, Raja Biswas

**Affiliations:** 1 Amrita Vishwa Vidyapeetham, Amrita Institute of Medical Sciences, Department of Microbiology, Ponekara, Kochi, Kerala, India.; 2 Amrita Vishwa Vidyapeetham, Center for Nanoscience and Molecular Medicine, Ponekara, Kochi , Kerala, India.; 3 Amrita Vishwa Vidyapeetham, Amrita Institute of Medical Sciences, Molecular biology laboratory, Ponekara, Kochi , Kerala, India.; 4 Amrita Vishwa Vidyapeetham, Amrita Institute of Medical Sciences, Department of Biostatistics, Ponekara, Kochi, Kerala, India.; 5 Amrita Vishwa Vidyapeetham, Amrita Institute of Medical Sciences, Department of Gastroenterology, Ponekara, Kochi, Kerala, India.

**Keywords:** Hepatitis C Virus core antigen, Hepatitis C Virus, RNA, HCV antibodies, Sustained viral response

## Abstract

**INTRODUCTION:**

We compared the hepatitis C virus (HCV) core antigen test with the HCV RNA assay to confirm anti-HCV results to determine whether the HCV core antigen test could be used as an alternative confirmatory test to the HCV RNA test.

**METHODS:**

Sera from 156 patients were analyzed for anti-HCV and HCV core antigen using a chemiluminescent microparticle immunoassay (Architect i2000SR) and for HCV RNA using the *artus HCV RG RT-PCR Kit* (QIAGEN) in a Rotor-Gene Q instrument.

**RESULTS:**

The diagnostic sensitivity, specificity, and positive and negative predictive values of the HCV core antigen assay compared to the HCV RNA test were 77.35%, 100%, 100%, and 89.38%, respectively. HCV core antigen levels showed a good correlation with those from HCV RNA quantification (*r =*0.872). However, 13 samples with a viral load of less than 4000 IU/mL were negative in the HCV core antigen assay. All gray-zone reactive samples were also RNA positive and were positive on repeat testing.

**CONCLUSIONS:**

The Architect HCV core antigen assay is highly specific and has an excellent positive predictive value. At the present level of sensitivity (77%), the study is still relevant in a low-income setting in which most of the HCV-positive patients would go undiagnosed, since HCV RNA testing is not available and/or not affordable. HCV core antigen testing can also help determine the true burden of infection in a population, considering the fact that almost 50% of the anti-HCV positive cases are negative for HCV RNA.

## INTRODUCTION

Hepatitis C virus (HCV) belongs to the family *Flaviviridae* and has a positive-strand RNA genome^1^. According to WHO estimates, 170 million people are infected with HCV worldwide, of which 25% will clear the infection and 75% will remain chronically infected^2^ ^-^ ^4^. Accurate diagnosis of active HCV infection has become more important since directly acting antivirals have achieved more than 95% cure rates in most populations^5^. The awareness of HCV infection among individuals in low-and middle-income countries is low due to limited access to diagnostic facilities. The diagnostic algorithm for the diagnosis of HCV infection involves a screening test for anti-HCV antibodies (anti-HCV) by chemiluminescence immunoassay (CLIA) or enzyme immunoassay (EIA), followed by confirmation by HCV RNA testing in antibody-positive patients. HCV EIA testing has evolved from a first generation test employing recombinant antigen c100-3 to the current third-generation test using antigens from the NS5, core, and NS3 regions^6^. The major limitations of anti-HCV testing are its inability to differentiate between acute (on-going), past (resolved), and persistent (chronic) infection and its poor sensitivity in the early treatment window, the first 4-6 weeks of infection^7^. Moreover, even third-generation anti-HCV tests yield false positive or indeterminate results at rates of 11% or higher^8^ ^,^ ^9^. 

To confirm HCV infection or to establish clearance of virus after therapy, the National Institute of Health recommends the use of a qualitative HCV RNA test with a sensitivity of 50 IU/mL or less^10^. Although HCV RNA testing is a reliable method, it is time-consuming, expensive, and requires a laboratory equipped with personnel with appropriate technical skills. The sequence of HCV core protein (HCV Ag), a structural protein, is highly conserved among all genotypes and quasispecies^11^. HCV Ag testing was developed as a cost-effective, sensitive, and specific assay that is easy to perform and could reduce the serological period for confirming HCV infection. It was first used to screen for anti-HCV-negative individuals in 1996^12^. Since then, it has been used extensively to diagnose active infection, assess chronic infection, and monitor response to treatment^13^. The HCV Ag test has several advantages compared to HCV RNA testing, including that its stability at room temperature allows for unrefrigerated transport, it correlates well with HCV RNA levels, and can use the same platform that is used for anti-HCV testing. HCV Ag test has a detection lower limit of 3000-10000 IU/mL of HCV RNA, which is less sensitive compared to PCR assays, which have a lower limit of 12-15 IU/mL of HCV RNA^13^ ^,^ ^14^. Studies on the HCV Ag test have reported a sensitivity of >90% and a specificity of >98% for the diagnosis of HCV infection^13^.

This study compared the HCV Ag test with the HCV RNA assay to determine whether the HCV Ag test can be used as an alternative to the HCV RNA test for confirming HCV infection. It also compared the diagnostic sensitivity and specificity of the HCV Ag test with the HCV RNA test.

## METHODS

The Architect i2000SR system (Abbott Diagnostics, IL, USA) was used to screen 156 consecutive plasma samples for HCV using an anti-HCV assay, which uses NS3 and NS4 proteins and c100-3 to detect antibodies by chemiluminescent microparticle immunoassay (CMIA). The Architect i2000SR system was also used for the HCV Ag assay, which quantifies HCV Ag using a two-step CMIA in human serum or plasma samples. The method a lower detection cut-off at 3 fmol/L (0.06 pg/mL); therefore, samples with <3 fmol/L were considered undetectable while those with ≥ 3 fmol/liter were considered positive. Samples with levels from ≥ 3 to < 10 fmol/L were considered gray-zone reactive and tested in duplicate. If both values were ≥ 3 fmol/L, the sample was considered a positive reaction.

HCV RNA was isolated from plasma samples using the QIAamp MinElute Virus Spin kit (QIAGEN Inc., Germany) according to the manufacturer’s instructions. Quantitative diagnosis was performed using the QIAGEN artus HCV RG RT-PCR Kit in a Rotor-Gene Q instrument. The limit of detection of the kit is 34 IU/mL; levels above this are considered positive and lower values are reported as undetectable, since reproducibility of the result is not assured.

Statistical analysis was performed using IBM SPSS software (version 20.0). Categorical variables are expressed as frequencies and percentages. Numerical variables are presented using mean ± standard deviation. Diagnostic measures such as sensitivity, specificity, positive predictive value, negative predictive value, and accuracy were calculated. Kappa statistics were used to compare the agreement between HCV antigen and HCV RNA. Pearson’s correlation coefficient was used to assess the relationship between HCV core antigen and HCV RNA. Linear regression analysis was used to predict HCV antigen levels using HCV RNA as a predictor variable. P-values <0.05% were considered significant.

## RESULTS

Among the 55 (35.25%, 55/156) HCV RNA-positive samples, 76.36% (42/55) were also positive for HCV Ag (Table 1). HCV Ag tests were negative in 23.63% (13/55) of samples that were positive for both anti-HCV and HCV RNA. Of the 39 anti-HCV-negative samples one tested positive for both HCV Ag and HCV RNA and one tested positive only for HCV RNA. The overall sensitivity and specificity of HCV Ag for confirming viremia were 77.35% (95% CI, 86%-98%) and 100% (95% CI, 86%-98%), respectively (Table 2). The 6 HCV Ag gray zone reactive samples had a viral load ranging from 350-8090525 IU/mL (Table 3). Of the 13 samples that were false negatives for HCV Ag, 4 had HCV RNA levels <500 IU/mL, 2 had 500-1000 IU/mL, 2 had 1000-2000 IU/mL, 4 had 2000-4000 IU/mL, and 1 patient had a very high viral load of 1,323,065 IU/mL.

**TABLE 1:** Summary of HCV antigen, HCV RNA and anti HCV test results.

HCV RNA (number of anti-HCV positive samples/total number)			
HCV Ag	Not detected	Detected	Total
Not detected	101 (62/101)	13 (12/13)	114 (74/114)
Reactive	0 (0/0)	42 (41/42)	42 (41/42)
**Total**	**101 (62/101)**	**55 (53/55)**	**156 (115/156)**

**TABLE 2:** Performance of the HCV Ag assay compared to the HCV RNA test.

	Sensitivity (%)	Specificity (%)	PPV (%)	NPV (%)	Accuracy (%)
**HCV Ag**	77.35%	100%	100%	89.38%	92.20%
	(62.98 - 86.77%)	(96.41 - 100%)		(82.85 - 92.59%)	(86.17 - 95.49)
**HCV Ab**	96.36%	38.61%	46%	95.1%	58.97%
	(87.47 - 99.56%)		(42.72 - 51.13%)	(83.75 - 98.79%)	(52.12 - 67.99%)

Values in parentheses are ranges; **PPV:** positive predictive value; **NPV:** negative predictive value.

**TABLE 3:** Summary of samples that were gray-zone reactive for HCV antigen assay.

Sample	HCV RNA (IU/mL)	Anti-HCV	HCV Ag 1^st^ assay	HCV Ag 2^nd^ assay
1	47739.65	23.4	8.39	24.92
2	3202.9	13.9	7.21	11.39
3	350	11.16	6.56	4.18
4	4932.325	12.78	7.46	4.18
5	18385.65	12.58	9.77	7.57
6	8090525	8.69	7.06	5.77

Anti-HCV S/Co > 1 was considered reactive; HCV Ag ≥ 3 fmol/L but < 10 fmol/L were considered gray-zone reactive.

The only sample that tested positive for HCV RNA but negative for both HCV Ag and anti-HCV had an RNA concentration of 70.7 IU/mL. The patient was not on any antiviral treatment and was a window-period infection case. The sample that was a false negative for anti-HCV had an infection of the 1a/1b HCV genotype, with a very high viral load of 15,210,031 IU/mL and high HCV Ag level of 20000 fmol/L. He was treated with directly acting antivirals (sofosbuvir/velpatasvir combination) for 12 weeks. At 24 weeks, his viral load was <33.63 IU/mL, and the HCV Ag and anti-HCV tests were negative. With 1 exception, HCV Ag tests were positive for all samples with an HCV RNA concentration >4000 IU/mL; the lowest HCV RNA concentration that was positive for HCV Ag was 350 IU/mL. All but 1 of the anti-HCV results were positive when the HCV Ag test was positive. The positive and negative predictive values and accuracy of the HCV Ag test compared to the HCV RNA test were 100%, 89.38%, and 92.20%, respectively. The concentrations of HCV Ag and HCV RNA were consistent throughout the common dynamic range of the assay.

## DISCUSSION

The detection of anti-HCV using CLIA or EIA is the common method for diagnosis of HCV infection worldwide. The biggest limitation of the anti-HCV assay is its false positivity rate at low titers. False-positive results have been reported to be as high as 35% in areas with an HCV prevalence of <10%^1^ ^,^ ^15^ ^,^ ^16^. It is noted that only 47% (55/117) of the anti-HCV-positive samples were HCV RNA-positive in this study. 

The disadvantages of the anti-HCV test include a window period of 45-68 days before the appearance of detectable antibodies, false negative results in immunocompromised patients, and the inability to distinguish past, acute, or persistent infection from each other^7^. Though we identified 1 case with a window period infection, the seroconversion could not be documented, since he was lost to follow-up. In patients with resolved infection, anti-HCV tests might remain positive for a prolonged period or for life, making it impossible to differentiate from active/ongoing infection. Therefore, anti-HCV-positive samples should be confirmed by HCV RNA testing or pre-confirmatory HCV Ag assay^10^. 

HCV RNA assays used to confirm HCV infection in anti-HCV-positive patients are performed by amplification methods such as real-time PCR or signal amplification by branched DNA and transcription-mediated amplification, which are prohibitively expensive and require highly trained personnel to operate the technical equipment. HCV Ag is a marker of HCV replication and can be used as an alternative to RNA for the detection and diagnosis of acute or chronic HCV infection. HCV Ag testing has been recommended by the 2017 WHO Global Hepatitis Report and 2018 European Association for the Study of Liver (EASL) guidelines as an alternative option when HCV RNA testing is not available and/or is not affordable^17^ ^,^ ^18^. The recommendation for HCV Ag testing was based on its high sensitivity and specificity rates, cost-effectiveness, ease of performance, and ability to diagnose in the early window period. However, the HCV Ag test is less sensitive than the HCV RNA assay and depends on the HCV genotype. Previous studies reported that HCV Ag levels of the 1b genotype were higher than those of the 3b, 2, and 1b/3b mix genotypes^19^ ^,^ ^20^. Even though HCV RNA real-time PCR was available in our institute, it was performed only once a week after pooling samples to make it cost-effective, whereas the HCV Ag assay could be performed at any time of the day after the instrument was calibrated. The performance time of the HCV Ag assay is 36 min, while HCV RNA real-time PCR requires 4 h.

In the present study, the HCV Ag Architect CMIA had sensitivity, specificity, and positive and negative predictive values of 77.35%, 100%, 100%, and 89.38%, respectively. Since these values vary with the prevalence of HCV in a given population, they cannot be generalized. Compared with previously published studies that used the same Architect HCV Ag assay, the diagnostic specificity of 100% in our study is equal to the rates reported by Kesli et al.^7^,Florea et al.^21^, Buket et al.^22^, Chevaliez et al.^23^, Park et al.^24^and Ross et al.^25^ and almost equal to values reported by Morota et al.^26^ (99.8%), Miedouge et al.^27^ (99.2%), and Leary et al.^28^ (99%). However, the 77.35% diagnostic sensitivity was lower than the rates reported by most previous studies, including Chevaliez et al.^23^ (98.11%), Kesli et al.^7^ (96.3%), Song et al.^29^ (97.2%), and Park et al.^24^ (90.2%), but higher than the rates reported by some, including Ergunay et al.^30^ (72.40%), Florea et al.^21^ (74.14%), and Gu et al.^31^ (43.63%). A previous meta-analysis found that the Architect HCV Ag assay had an overall sensitivity of 93.4% and specificity of 98.8%. The low sensitivity of 77.35% in this study might be attributed to the low seroprevalence of anti-HCV in sample population (0.64%, unpublished report), and to the small sample size. In this study, 19% of the samples would have required HCV RNA testing to confirm active infection if the HCV Ag assay was used as the second-line test. 

Although HCV Ag identified all cases with a viral load > 4000 IU/mL, it tested negative in a solitary case with a very high viral load of 1323065 IU/mL. A similar case was reported previously, where the baseline HCV Ag test was negative in a patient with genotype 1 infection and a high HCV RNA concentration of >2000000 IU/mL^32^. It should be noted that the HCV Ag assay missed 1 case of window-period infection in our study. Although gray-zone HCV Ag results need to be retested, resulting in additional cost and time, none of our 6 gray-zone reactive samples were false positives (RNA positive/antibody positive). On retesting, none of them tested negative. Previous studies have shown that gray-zone results found to be negative on retesting do not require additional confirmatory testing^33^.

Due to the cost effectiveness and ease of performing the HCV Ag assay, HCV RNA testing might be reserved for samples positive for anti-HCV but negative for HCV Ag in low-income countries with a high incidence of HCV. Previous studies have shown that such an algorithm saves $0.29/individual tested^32^. The introduction of directly acting antivirals replaced the need for repeated viral load testing with testing prior to therapy and 12-24 weeks after the end of treatment. Since HCV Ag levels correlate with HCV RNA levels, undetectable HCV Ag in serum or plasma at 24 weeks after the end of treatment might be used as an alternative endpoint of therapy when considering sustained viral response in patients with detectable HCV Ag prior to therapy.

The main limitation of this study was its small sample size; its strength was the inclusion of an adequate number of true positives (35%), true negatives (25%), and false-positive samples (40%). This is the third study from India evaluating the usefulness of the HCV Ag assay for diagnosing HCV infection. The previous two studies evaluated HCV Ag assays from Ortho-Clinical Diagnostics (Rochester, New York)^34^ ^,^ ^35^. Due to a lack of genotyping in our study, the correlation between HCV Ag levels and genotypes could not be evaluated. At the present level of sensitivity (77%), the study is still relevant for low-income settings where most anti-HCV-positive patients would remain undiagnosed, since HCV RNA testing is not available and/or not affordable. HCV Ag testing can also help determine the true burden of infection in a population, considering the fact that almost 50% of the anti-HCV-positive cases test negative for HCV RNA. Due to the high degree of concordance between the HCV Ag levels and viral load, HCV Ag can be used for assessing the sustained viral response in patients being treated with directly acting antivirals and thereby reduce the overall cost of treatment.
